# The Role of Family-Level Factors in Firearm Storage Practices

**DOI:** 10.1007/s10900-025-01459-5

**Published:** 2025-04-11

**Authors:** Alexander J. Rice, Christin M. Ogle, Joscelyn E. Fisher, Stephen J. Cozza

**Affiliations:** 1 Department of Psychiatry, Uniformed Services University of the Health Sciences (USU), Bethesda, MD USA; 2 Henry M. Jackson Foundation for the Advancement of Military Medicine, Inc., Bethesda, USA

**Keywords:** Firearm storage, Family, Safety, Injury prevention, Decision-making, Public health intervention

## Abstract

Firearm-related injuries and fatalities among youth in the United States represent a critical public health crisis. Secure firearm storage (i.e., keeping guns unloaded, locked, and stored separately from ammunition) is a proven strategy to reduce these risks. However, many households do not consistently adopt these practices. This review examines four key family-level factors that influence firearm storage decisions: (a) early firearm socialization, (b) family decision-making dynamics, (c) other household safety practices, and (d) parent understanding of child development and motivations regarding storage. Findings indicate that interventions may benefit from empowering parents to reflect on their early firearm socialization experiences, improving their understanding of children’s development, integrating firearm safety into broader household safety frameworks, and promoting collaborative decision-making in multi-adult households. Future research should further investigate how these factors intersect to shape firearm storage practices, including the long-term effects of early firearm exposure through longitudinal studies, and variations across diverse family structures and contexts, including multigenerational households.

Firearm-related injuries and fatalities remain a significant public health crisis in the United States (US), profoundly impacting children and adolescents. In 2022, firearms surpassed all other causes as the leading source of death among youth aged 1–17, claiming an average of seven lives per day [1]. This trend represents a doubling of youth gun deaths since 2013, driven by both homicide and suicide [1]. Beyond the loss of life, firearm violence imposes lasting consequences on survivors and communities, including non-fatal injury, psychological trauma, poor academic and occupational outcomes, and increased risks of substance use and self-harm [2–5]. These outcomes underscore the urgent need to adopt comprehensive strategies to mitigate firearm-related harm, particularly within the home where youth can most readily obtain firearms.

Secure firearm storage, referring to concrete measures like storing firearms unloaded, locked, and separate from ammunition (as opposed to the more subjective term “safe storage”), is an effective method for reducing firearm-related injuries and fatalities in households with children and adolescents [6, 7]. Secure storage can prevent access to firearms or create delays critical to disrupting impulsive actions, such as suicide attempts. Indeed, research consistently links secure firearm storage to reduced risk of unintentional injury, homicide victimization, and suicide [6, 8, 9]. Family household storage, in particular, plays a pivotal role. An estimated 79% of adolescent firearm suicides and 41% of school shootings involve a family member’s firearm, while firearms used in unintentional injury deaths are most commonly accessed from nightstands or other sleeping areas (30%) within the home while unsecured [7, 10, 11]. 

Despite numerous legislative and programmatic efforts promoting secure firearm storage in the home (e.g., Child Access Protection or CAP laws) and broad endorsement of these practices by firearm owner advocacy groups (e.g., the National Rifle Association; the National Sports Shooting Foundation) [12, 13], non-secure storage practices remain widespread in U.S. households. For example, studies indicate that 20–43% of firearm-owning households with children store at least one firearm unlocked and loaded [14–16]. The persistence of less secure family practices, despite their known risks and the availability of widely-endorsed solutions, underscores the importance of understanding how family-level factors shape household storage decisions and practices.

While existing research on firearm storage has tended to focus on individual and community characteristics [16, 17], emerging evidence highlights the role of family-level influences on storage behaviors. For example, family discussions have been cited as an important factor in firearm storage decisions by gun owners [18]. Further, owners whose storage decisions were reportedly influenced by family discussions had 39% higher odds of storing their firearms locked [18]. 

This review examines four critical family-level influences on family firearm storage decisions that hold promise for modification through interventions: (a) early firearm socialization, (b) family decision-making dynamics, (c) other household safety practices, and (d) parental understanding of child development and motivations regarding storage. Firearm storage practices often reflect the early firearm socialization experiences that family members bring to these decisions, the ways in which decisions are negotiated within households, and how firearm safety is integrated with broader household safety practices. In families with children, these decisions are further influenced by how adult family members understand and respond to children’s behaviors and motivations as they relate to storage practices. Note that this review uses the term “parent” to refer to adult caregivers/family decision-makers, reflecting the focus of much of the research on parental relationships. However, where this term is used, the findings may also extend to other caregiving relationships, such as grandparents, guardians, or other adults in caregiving roles. Given the critical role families play in preventing youth firearm-related injuries and fatalities, interventions addressing these four family-level factors could motivate families to prioritize safety and reduce firearm-related harm. While this review focuses on family-level influences, known individual, household, and community risk factors provide essential context for understanding how family storage practices emerge and are examined in the next section.

## Individual, Household, and Community Characteristics Associated with Firearm Storage

Individual factors associated with less secure storage practices include conservative political affiliation, formal firearm training, larger numbers of firearms, handgun ownership, protection as an ownership motivation, and heightened threat perceptions [18–21]. In studies of households with children, individual factors such as handgun ownership and protection as an ownership motivation were linked with non-secure storage, whereas political affiliation was not [16]. 

Research has also explored the relationship between military service and firearm storage practices. In three studies, active or 

past military service was not significantly associated with storage practices [18, 22, 23]. However, one study found that active or past military service was linked to greater likelihood of storing loaded firearms in a drawer [20]. 

Concerning household factors, presence of children in the home was linked to more secure firearm storage practices, potentially driven by heightened awareness of the risks associated with firearm access by children [18, 22, 23]. However, households with older children tend to adopt less secure storage practices than those with younger children, which may reflect misplaced trust in older children’s judgment or a perceived need for them to access firearms in certain situations [15, 16, 24]. Findings on the association between income and firearm storage were inconsistent: two studies linked higher income to greater storage security [20, 22], whereas one study in families with children did not find a significant association [16]. 

Community norms and environments, including geographic regional differences, degree of urbanization, and public policies, have also been linked with family firearm storage decisions. For instance, Southern households often adopt less secure practices compared to those in other regions of the US [22, 23]. Evidence on urban versus rural settings is mixed: while three studies found no difference in secure storage [16, 18], one study reported that urban households were less likely to use gun safes than rural households [20]. Additionally, farm households were associated with less secure storage compared to non-farm rural households [25]. Statewide policies like CAP laws have been associated with reduced nonfatal firearm injuries, but have shown limited impact on storage behaviors, likely due to inconsistent enforcement and low awareness of their existence among the public [26, 27]. 

Although many individual, household, and community factors are associated with firearm storage behaviors, these decisions are expected to be negotiated within a family context. Each family member brings individual characteristics, motivations, and experiences shaped by community norms and environments to the family’s decision-making process. In addition, family-specific behaviors, perceptions, and relationships are likely to further influence storage decisions. Understanding how these factors interact within familial contexts to shape household practices is essential for developing effective safety interventions. Although some work has been conducted in this area, further attention to family-level factors influencing firearm storage practices is required.

## Family-Level Factors

Four key family-level factors—early firearm socialization, family decision-making dynamics, other household safety practices, and parental understanding of child development and motivations regarding storage—offer critical insights into how families perceive and manage firearm-related risks. The following sections examine each factor in detail, providing a deeper understanding of how these factors may influence firearm storage decisions within families and offering potential pathways for future prevention strategies.

**Early firearm socialization.** Firearm socialization reflects how early experiences with firearms shape attitudes and behaviors [28]. Positive early firearm experiences, including recreational activities like hunting, which may normalize the presence of firearms in the home, are associated with attitudes that prioritize accessibility over safety [28–30]. Conversely, negative firearm experiences, such as witnessing threats or injuries, are associated with attitudes that emphasize safety and restricted access [28]. These early experiences may also influence storage practices. For example, individuals who have experienced physical victimization were found to be more likely to engage in less secure firearm storage behaviors [29]. 

Experiences in one’s family of origin in particular may be significant in shaping storage behaviors. Similar to the intergenerational transmission of health-risk behaviors such as tobacco or alcohol use, normalization of certain firearm storage practices during childhood may perpetuate those practices in adulthood [31, 32]. For instance, families may rationalize non-secure storage practices if firearms were historically stored unlocked and loaded without incident [29]. A key mechanism of intergenerational firearm socialization may be parental modeling, which was found to be a stronger predictor of children’s intended future safety behaviors than parental rules or verbal instructions [33]. Emerging findings on storage practices indicate that individuals raised in firearm-owning households, in addition to those who experienced past physical victimization, are more likely to engage in less secure storage behaviors [20]. These findings, while preliminary and based on retrospective designs, underscore the potential influence of early firearm socialization on adult storage attitudes and behaviors.

**Family decision-making dynamics.** Family decision-making dynamics influence how each family member’s perspective, shaped by factors such as socialization, inform firearm storage practices. In many two-parent households, one parent, often the firearm owner, assumes primary responsibility for storage decisions, while the other may be excluded or defer [34, 35]. Males in particular, may exert greater influence on household firearm storage decisions, given their higher rates of firearm ownership and implicit or explicit recognition of their assumed expertise as firearm owners [36]. Given that male firearm owners tend to be more likely to allow children unsupervised access to firearms than women, family decision-making that does not include the perspective of a firearm owner’s partner may lead to less safe firearm practices, including use of less secure storage methods [19]. 

Excluding non-owning partners from firearm storage decisions can have significant safety repercussions, as these partners may be unaware of unsafe practices and unable to advocate for more secure storage measures [37]. Similarly, excluding parents from these discussions limits opportunities to align on storage practices and build a shared sense of responsibility for firearm safety [38]. Although parents report that avoiding open communication about firearm storage is relatively uncommon, they may be unaware of power imbalances that undermine secure storage practices [38]. Notably, families in which both partners actively participate in firearm storage decisions and share responsibility for safety measures are more likely to adopt secure storage practices [38]. 

Partner decision-making around firearm storage has been categorized into three relational patterns: collaborative, deferential, and devalued [35]. Collaborative relationships, marked by mutual respect and shared responsibility, are most strongly associated with responsive, safety-oriented decision-making [35]. In these relationships, firearm-owning partners were more open to improving storage practices, particularly when children or grandchildren were present. In deferential relationships one partner’s preferences took precedence, but the deferring partner felt empowered to voice concerns and act if they perceived a need. In contrast, devalued partners were discouraged from participating in decisions altogether. Lack of participation by one or more decision-makers hinders safety improvements and reinforces power imbalances in decision-making [35]. These findings highlight the critical role of family decision-making dynamics in shaping firearm storage practices and emphasize the need for interventions that foster collaboration and shared responsibility.

**Other household safety practices.** Parents’ firearm storage decisions may reflect their broader adherence to household safety practices. Studies have found similar adherence across household safety practices and identified shared factors influencing safety behaviors, such as knowledge of risks, perceived severity and controllability of harm, understanding of child development, and social influences [39–42]. When parents perceive similar vulnerability across household hazards and believe safety practices uniformly reduce risks, they may be more likely to implement consistent safety behaviors, including firearm storage practices [42]. Safety practices may align most when the risks they address share characteristics, such as controllability, a child’s likelihood of interacting with the hazard, or the severity of potential harmful outcomes. For instance, parents who recognize both pools and firearms as severe risks that can be mitigated through specific precautions may adopt safety measures of comparable rigor. However, studies have not examined potential influences on the relationship between firearm storage and other safety measures.

Evidence regarding the relationship between firearm storage and other household safety practices is limited, though some studies suggest potential connections. For instance, Martin-Storey et al. [43] found that families with inconsistent helmet use were more likely to store firearms less securely, indicating overlap between these two safety behaviors. However, the same study reported no significant association between firearm storage and other safety measures, such as consistent car seat use or functional smoke detectors, suggesting weaker alignment with those practices. Similarly, Coyne-Beasley et al. [37] did not identify a significant link between firearm storage and other home safety practices. Notably, these authors’ use of a composite measure encompassing seven safety practices may have obscured associations with specific behaviors. Taken together, the observed link between helmet use and firearm storage, supported by a conceptual framework of shared determinants of safety behaviors, suggests that framing firearm storage as one component of a family’s broader safety approach could be a promising strategy to improve firearm storage practices.

**Parental understanding of child development and motivations.** In families with children, another potential influence on family storage decisions is parents’ understanding of their children’s development and motivations with respect to firearms. Parents tend to overestimate their children’s developmental capabilities with respect to firearms, which likely compromises the secure storage of firearms. For example, while 66% of parents of children aged 7 to 17 believed their child could distinguish a toy gun from a real gun, only 41% of children demonstrated this ability [44]. Similarly, many parents perceive their own children to be at lower risk for firearm incidents than their peers, reflecting a bias that reduces the likelihood of adopting effective storage methods [45]. 

Misperceptions of developmental variations in children’s behavior may also undermine firearm storage decisions. For example, 97% of parents with children aged 5 to 15 years expected their children to react identically when encountering a firearm even when those children were of different ages, overlooking well-documented age-related differences in brain regions associated with risk-taking and inhibition [46]. This uniformity of perspective may lead parents to underestimate the need for secure storage practices that are appropriate to the developmental stages of the children in the household.

These misperceptions can extend to children’s motivations and impulses. Parents often believe that teaching firearm safety rules or modeling safe firearm handling will demystify firearms and deter unsafe behaviors, overlooking the influence of motivations and impulses [45]. In one study, over half of parents believed their children would avoid touching a firearm because they were “too smart” or “[knew] better” [46]. However, research using simulated scenarios shows that even children who can accurately articulate firearm safety rules frequently handle firearms when unsupervised and even do so in an unsafe manner, such as by pulling the trigger [47, 48]. These behaviors likely stem from well-documented developmental traits, such as curiosity, novelty-seeking, and impulsivity, which can override learned rules in the absence of supervision.

Educating children about firearm safety has limited impact on the likelihood that they will handle firearms unsupervised [49]. Even a recent clinical trial that demonstrated some effectiveness of a gun safety training video among 8–12 year old children, found that shortly after the video, 39.3% touched a gun while unsupervised and 8.9% pulled the trigger [50]. Educating their children about firearm safety may unfortunately thus provide many parents with a false sense of security regarding their children’s firearm access, leading them to underestimate the importance of secure storage. Indeed, parents who taught their children firearm handling and shooting were more likely to store firearms unsecured and loaded compared to those who did not provide such instruction [51]. 

Together, these misperceptions may account for the tendency of parents to underestimate their children’s ability to locate and access firearms within the home. For instance, 39% of children contradicted their parents’ claims that firearms were hidden or inaccessible, and 22% reported having handled a household firearm despite parental reports to the contrary [15, 44]. In households where parents believed their teenager could not access a firearm, 37% of children reported being able to access one within an hour, including 22% who reported access within five minutes [15]. Although these findings do not directly assess the role of parental perceptions in firearm storage decisions, they reveal a significant disconnect between parental beliefs and children’s behaviors that likely impact storage choices. Addressing this disconnect by aligning parental perceptions with developmental realities and emphasizing secure firearm storage practices that reliably limit child access is essential for reducing household risks.

## Discussion

Firearm-related injuries and fatalities among youth represent a critical and preventable public health issue, with families playing a central role in mitigating these risks. This review identified four family-level factors that shape firearm storage behaviors and present opportunities for intervention. Addressing these factors offers a path to more effective, yet undeveloped strategies for promoting secure firearm storage and reducing firearm-related tragedies. The following discussion explores the implications of these findings, highlighting how each factor can inform interventions and guide future research.

Interventions could empower parents to make informed decisions by illuminating how firearm socialization experiences, such as family of origin storage practices, participation in firearm-related activities, and harmful or potentially harmful incidents during childhood, can influence whether accessibility or security is prioritized. Encouraging parents to critically evaluate how these early experiences inform their current storage decisions could highlight early life norms that do not align with contemporary safety priorities and circumstances. By encouraging this reflection and reminding firearm owners that they may choose differently than how they were raised, interventions can support thoughtful and contextually appropriate storage decisions while recognizing the potential emotional and cultural significance of these norms.

Moreover, household safety behaviors, despite mixed evidence on their relationship with firearm storage practices, offer valuable insights into family approaches to safety that are relevant to firearm safety. Variations in these practices likely reflect how families perceive and prioritize risks, as well as the influence of external factors such as cultural or community norms, which may uniquely shape firearm storage decisions. Contextualizing firearm safety as a part of broader household safety decisions could encourage families to practice consistent safety behaviors in response to a wide range of household risks, including secure firearm storage. Positioning firearm safety as part of a comprehensive family safety strategy could normalize these behaviors and reduce resistance to storage discussions due to firearm-specific concerns such as ownership rights.

Parents can also benefit from examining their perceptions of their children’s ability to stay safe in the presence of firearms and how these perceptions align with developmental realities. For example, education could highlight how curiosity, impulsivity, and the tendency to explore novel items might lead children to access firearms despite firearm safety instruction. It may be helpful to remind parents that the risks they themselves often took in childhood may be similar to those they now assume their own children are either incapable of or not willing to take. Simulated scenarios or reflective exercises could help parents recognize the importance of secure storage by illustrating children’s behavioral tendencies. Additionally, helping parents understand how developmental trends, such as increasing independence and risk-taking during adolescence [52], affect firearm-related risks may encourage more consistent storage practices over time.

Finally, interventions could encourage parents to explore their decision-making dynamics around firearms. Findings highlight how relational dynamics within families can shape firearm storage practices. Collaborative decision-making between parents is associated with more secure practices, while inequities, such as one partner dominating decisions or excluding the other, can undermine those efforts. Interventions could foster collaboration by addressing relational barriers, such as differing priorities for firearm access or misaligned perceptions of risk. Helping families identify shared safety goals and providing tools for equitable conversations about storage practices could reduce relational barriers and encourage sustained adherence to secure storage.

Importantly, although discussed individually, these family-level factors are likely interconnected in practice. For instance, decision-making dynamics can shape how parental understanding of child development translates into specific storage practices or whether socialized firearm norms are adopted or challenged. Recognizing these relationships, along with their relationship to broader individual, household, and community influences, is essential for designing interventions that address the multifaceted nature of firearm storage decisions.

## Future Research

**Fig. 1:**
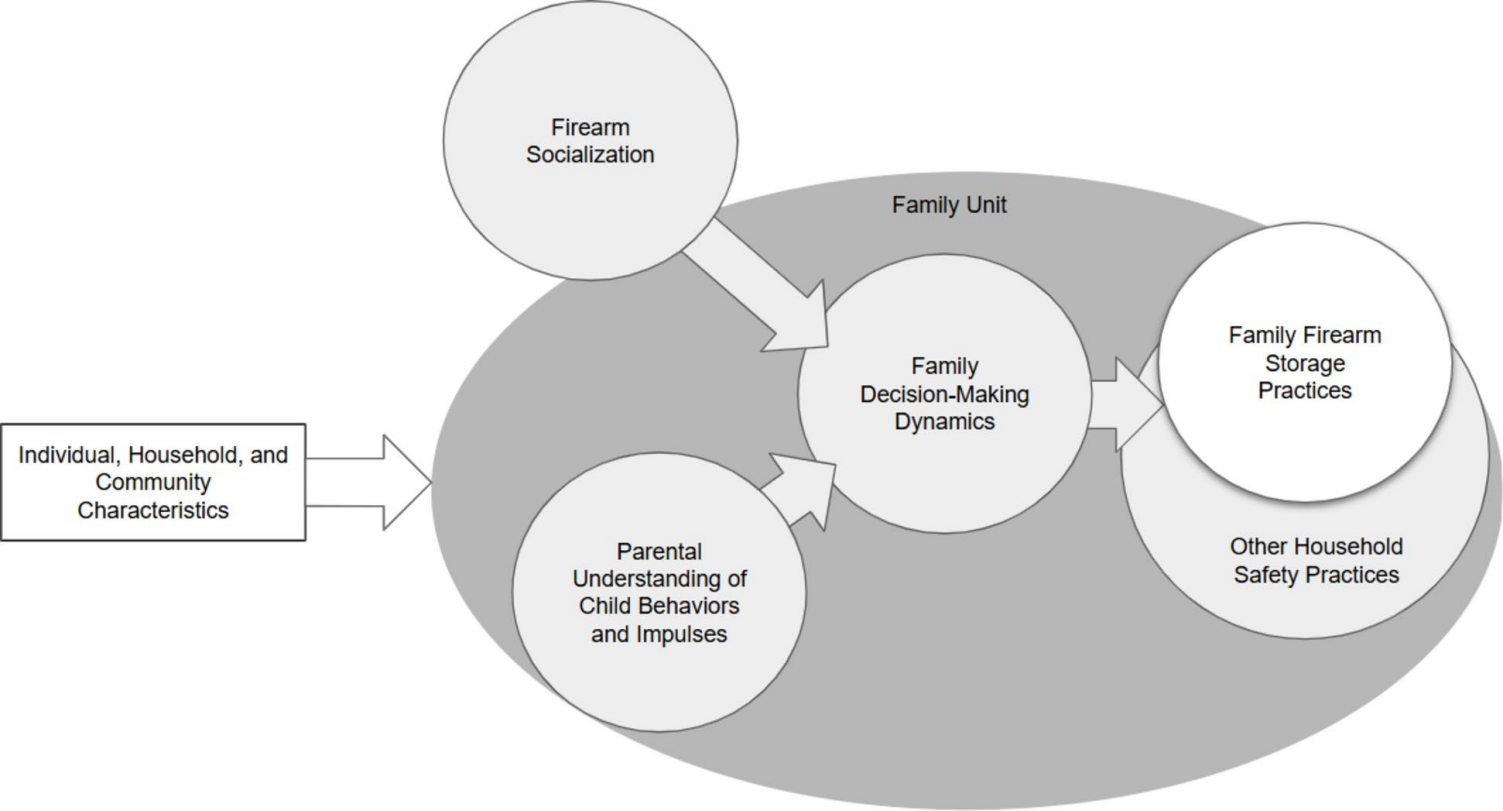
Family-level factors associated with firearm storage practices

Emerging evidence suggests that family-level factors play a significant role in shaping firearm storage decisions, but further research is needed to clarify their specific impacts and interactions. Developing and testing plausible models of how these factors interrelate could help identify where interventions may be most effective. For example, one potential model posits that firearm storage practices are a specific component of a family’s broader safety practices, shaped by parental decision-making dynamics. These dynamics, in turn, may be influenced by parents’ understanding of child development and motivations, as well as their socialization of firearm attitudes and behaviors (see Fig. 1).

Understanding how family socialization shapes perceptions of firearms relative to other safety practices may be particularly valuable for designing interventions. For example, families that view firearms as comparable to hazards like poisons may naturally integrate secure storage into their broader safety routines. In contrast, families that see firearms as tools for self-defense or symbols of autonomy may prioritize accessibility over safety. Interventions could help families critically examine the influence of socialization on these perceptions, framing secure firearm storage as a vital component of comprehensive household safety.

Understanding of how early exposure to firearm norms and behaviors shapes storage practices over time could further be enhanced by longitudinal studies that mitigate the retrospective recall bias inherent in current research on family firearm socialization. Such studies would offer valuable insights into how familial attitudes and behaviors evolve and influence long-term storage practices. Further, exploration of parental understanding of children’s developmental tendencies relevant to firearm storage, such as curiosity and impulsivity, is needed to determine whether direct links exist between these perceptions and storage decisions. Developing standardized measures to assess parental views on child safety-related motivations and impulses would be critical to this research. Future research should also examine whether alignment in parental views of child development and safety priorities strengthens collaboration when making firearm storage decisions and explore how specific mechanisms, such as communication styles, conflict resolution strategies, and power dynamics, impact family firearm storage practices.

Finally, examining how family-level factors vary across different family types and contexts could inform the development of tailored interventions, depending upon family culture, race, ethnicity, and make up. For example, the influence of family-level factors may differ in multigenerational households where older adults, such as grandparents, have different risk perceptions or safety priorities compared to parents. The role of family level factors in the context of shifting household circumstances, such as co-parenting in separate households, the presence of houseguests, changes in household roles, or new living arrangements, could also be examined. Understanding these variations could provide critical insights into the ways families adapt storage practices to fit their unique structures and circumstances, laying the groundwork for more flexible interventions.

## Conclusion

Integrating family-level factors into a comprehensive framework for understanding firearm storage practices offers a promising pathway to reducing firearm-related injuries and fatalities among youth. Targeted interventions that address these modifiable influences have the potential to empower families to adopt secure storage practices and mitigate firearm-related harm. This review highlights the urgent need for continued research and innovation to provide families with the knowledge, tools, and support necessary to create safer home environments and protect children from firearm-related risks.
